# Supplementary material for: Validation of Pharmacological Protocols for Targeted Inhibition of Canalicular MRP2 Activity in Hepatocytes Using [99mTc]mebrofenin Imaging in Rats
Source: Pharmaceutics. 2020 May 27;12(6):486. doi: 10.3390/pharmaceutics12060486 (PMC7355955; doi:10.3390/pharmaceutics12060486)
Supplement: Supplementary file 1 [file pharmaceutics-12-00486-s001.pdf]

pharmaceutics

MDPI

Supplementary Materials: Validation of Pharmacological Protocols for Targeted Inhibition of Canalicular MRP2 Activity in Hepatocytes Using [^{99m}Tc]mebrofenin Imaging in Rats

Solène Marie, Irene Hernández-Lozano, Louise Breuil, Wadad Saba, Anthony Novell, Jean-Luc Gennisson, Oliver Langer, Charles Truillet And Nicolas Tournier

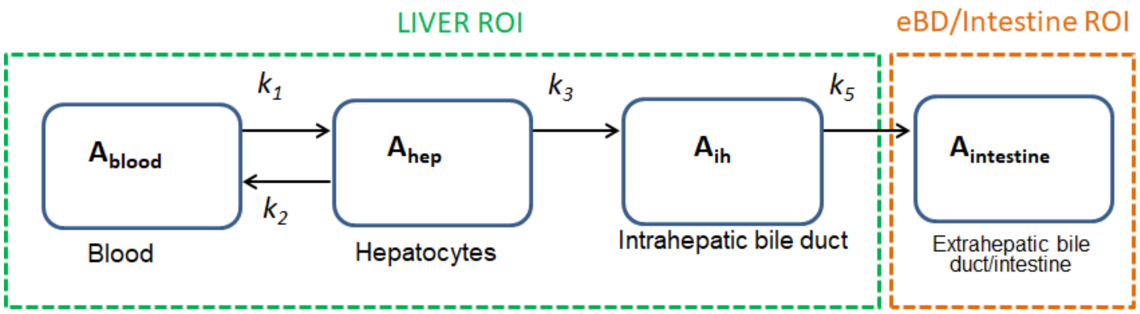

Figure S1. Four-compartment pharmacokinetic model. k_1 and k_2 describe the transfer of [^{99m}Tc]mebrofenin between blood and hepatocytes, k_3 from hepatocytes into the intrahepatic bile ducts and k_5 from the intrahepatic bile ducts to the intestine. ROI means Region of Interest, eBD means Extrahepatic bile duct.

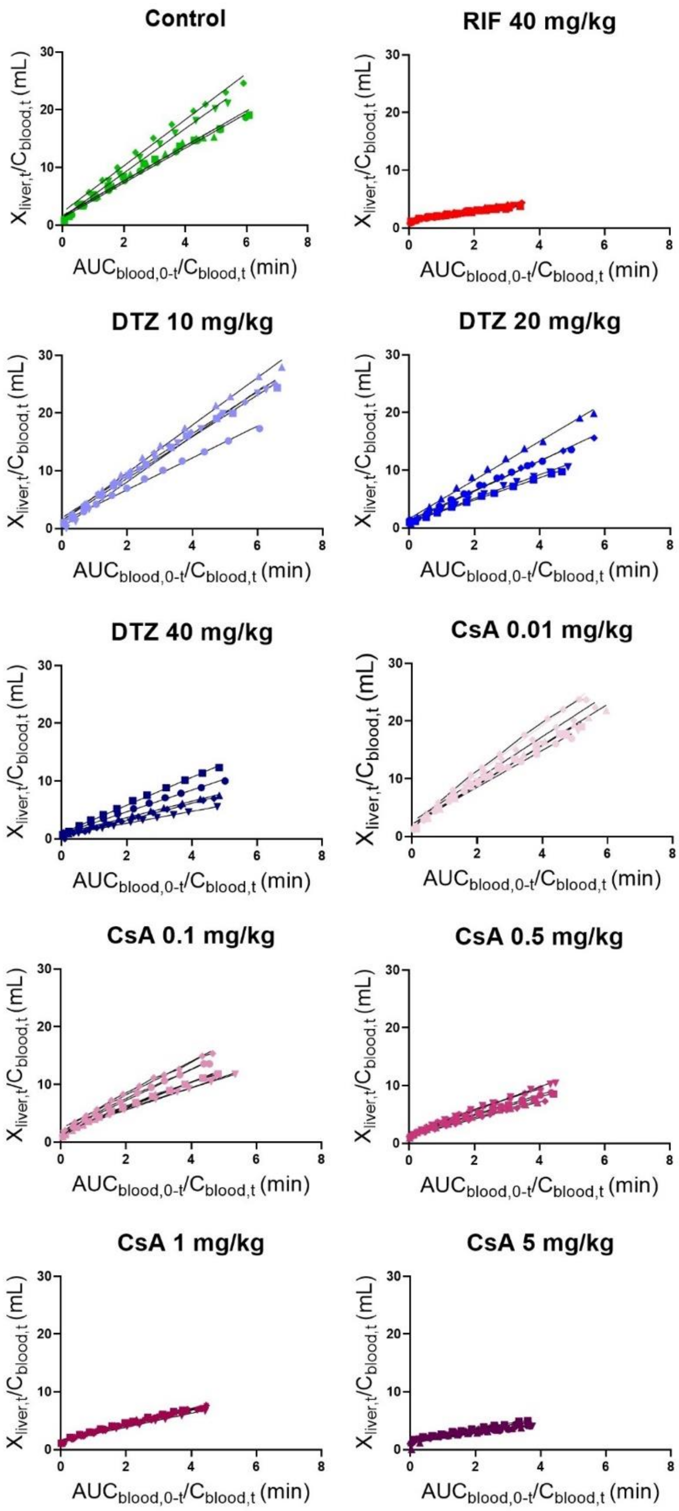

Figure S2. Integration plots for all investigated groups to estimate the uptake clearance of [^{99m}Tc]mebrofenin from blood into the liver for control animals and animals treated by rifampicin (RIF), diltiazem (DTZ) and cyclosporin A (CsA). CL_{uptake} and V_E correspond to the slope of the linear regression line and the Y-interception respectively.

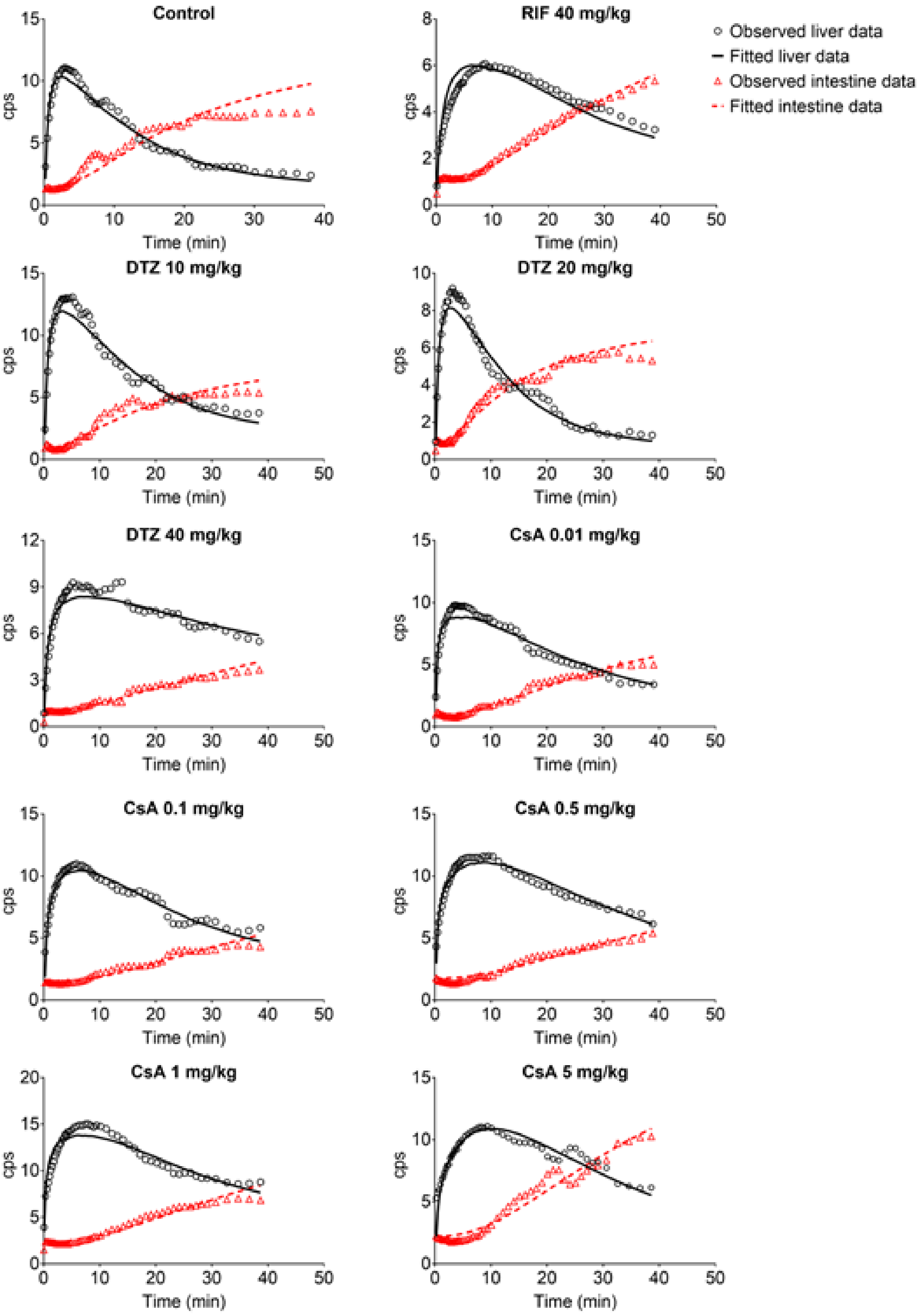

Figure S3. Time activity curves (cps: counts per second) of observed and fitted data in one representative subject of each study group for control animals and animals treated by rifampicin (RIF), diltiazem (DTZ) and cyclosporin A (CsA).

Table S1. Pharmacokinetic parameters obtained with the four-compartment model for the different study groups.

Group	k_1 (min^{-1})	k_2 (min^{-1})	k_3 (min^{-1})	k_5 (min^{-1})
Control	7.37 ± 1.62<br>(4.8 - 16.0)	0.15 ± 0.08<br>(7.2 - 30.8)	0.15 ± 0.08<br>(3.9 - 11.9)	0.12 ± 0.03<br>(2.4 - 12.5)
RIF (40mg/kg)	0.89 ± 0.09 *<br>(3.6 - 7.6)	0.01 ± 0.01 *<br>(28.7 - 125.7)	0.05 ± 0.02 *<br>(2.4 - 12.7)	0.64 ± 0.88<br>(13.6 - 31.7)
DTZ (10 mg/kg)	7.20 ± 0.90<br>(2.7 - 10.2)	0.10 ± 0.04<br>(3.1 - 16.2)	0.06 ± 0.04 *<br>(1.6 - 6.7)	0.22 ± 0.16<br>(4.1 - 15.9)
DTZ (20 mg/kg)	5.21 ± 1.35 *<br>(2.4 - 9.8)	0.06 ± 0.03 *<br>(3.6 - 25.8)	0.05 ± 0.01 *<br>(1.3 - 29.1)	0.31 ± 0.20<br>(5.2 - 27.8)
DTZ (40 mg/kg)	2.76 ± 0.8 9*<br>(2.6 - 10.2)	0.02 ± 0.01 *<br>(6.1 - 74.2)	0.06 ± 0.03 *<br>(1.9 - 10.9)	0.31 ± 0.24<br>(6.9 - 27.1)
CsA (0.01 mg/kg)	8.69 ± 1.90<br>(1.6 - 4.8)	0.09 ± 0.02<br>(3.1 - 7.9)	0.03 ± 0.01 *<br>(1.6 - 7.8)	0.34 ± 0.31<br>(6.2 - 18.5)
CsA (0.1 mg/kg)	5.43 ± 1.75<br>(1.2 - 6.3)	0.07 ± 0.02 *<br>(3.0 - 11.7)	0.03 ± 0.01 *<br>(2.9 - 11.4)	0.10 ± 0.05<br>(6.7 - 13.6)
CsA (0.5 mg/kg)	3.90 ± 0.67 *<br>(1.4 - 5.7)	0.05 ± 0.01 *<br>(3.6 - 17.7)	0.05 ± 0.03 *<br>(6.5 - 9.8)	0.09 ± 0.04<br>(5.8 - 19.3)
CsA (1 mg/kg)	3.73 ± 0.39 *<br>(2.2 - 4.6)	0.04 ± 0.02 *<br>(3.8 - 25.5.)	0.03 ± 0.02 *<br>(3.2 - 22.4)	0.13 ± 0.07<br>(5.7 - 21.9)
CsA (5 mg/kg)	2.81 ± 0.86 *<br>(2.2 - 10.6)	0.04 ± 0.02 *<br>(10.4 - 37.0)	0.15 ± 0.08<br>(6.8 - 37.6)	0.06 ± 0.02<br>(2.2 - 42.5)

Parameter values are given as the mean ± SD (n = 5 for control, RIF, DTZ 10 mg/kg, DTZ 20 mg/kg, DTZ 40 mg/kg, CsA 0.1 mg/kg groups and n = 6 for CsA 0.01 mg/kg, CsA 0.5 mg/kg, CsA 1 mg/kg, CsA 5 mg/kg groups). Values in parentheses express the range in percent coefficient of variation (%CV) of the parameters, which determines parameter precision. k_1 and k_2 are the rate constants for the transfer of [^{99m}Tc]mebrofenin between blood and liver tissue, k_3 is the transfer rate constant from liver tissue to the intrahepatic bile ducts, and k_5 is the transfer rate constant from the intrahepatic bile ducts to the extrahepatic biliary ducts and intestine. *$p < 0.05$, one-way ANOVA against a reference group (control) followed by a Bonferroni multiple-comparison test.
